# A low-cost color sensor device for rapid detection of high-grade serous ovarian carcinoma (HGSOC)

**DOI:** 10.18632/oncoscience.652

**Published:** 2026-03-11

**Authors:** Faisal Iqbal

**Affiliations:** ^1^Department of Pharmaceutical Sciences, University of Illinois Chicago, IL 60612, USA

**Keywords:** HGSOC, Pi, GUI, Ercose

## Abstract

A sensitive nucleic acid detection approach based on tracing the inorganic phosphate (Pi) created during amplification by means of the colorimetric method has been presented. This method relies on nucleic acid amplification. Pyrophosphate (PPi), a result of the nucleic acid polymerization reaction, was hydrolyzed into inorganic phosphate (Pi) by the addition of inorganic pyrophosphatase. To create the phosphomolybdate precipitate, the obtained Pi could react with acid molybdate. The color of amplified sample was changed into green. Using high grade serous ovarian carcinoma (HGSOC) as an example, this tactic’s usefulness was proven. Here, describe the Ercose (Eraser + Color Sensor) device proof-of-concept, which uses a straightforward strategy without sacrificing the accuracy of the outcomes. Ercose is a simple, quick, weightless and inexpensive. A low-cost, user-friendly color sensor is suggested for development in this study to identify the hue of the objects. The test objects were progressively illuminated by the sensor’s RGB light emitting diodes, which served as the light sources. The analogue voltage data were converted to digital form and serially delivered to a graphical user interface (GUI). To determine the test objects colors, the digitized voltage values were employed (i.e., RGB values). The microcontroller arduino nano was managed by the GUI, which also showed the needed color values. The Ercose device can be used in a variety of real-world scenarios where standard spectrophotometer devices are not practical.

## INTRODUCTION

Medical diagnosis has benefited greatly from nucleic acid assay, however it is constrained by the technique for amplified product detection. Amplification can be done using RPA, PCR, or LAMP, among other techniques. Modern techniques are now employed to track the amplification of target DNA, such as real-time thermal cyclers. The generation rate for one or more particular products can be determined using the thermal cycler’s sensor for measuring the fluorescence of the fluorophore after it has been stimulated at the necessary wavelength. This permits the generating rate for amplified products. The ability to illuminate each sample with a beam of light at least one defined wavelength and detect the fluorescence given off by an excited fluorophore is a feature of real-time thermal cyclers. In order to benefit from the physiochemical characteristics of nucleic acid, the thermal cycler can also quickly heat and cool the sample. Computer software can instantly analyze the data that is produced. The high expense of equipment like real-time thermal cyclers, dyes like SYBR Green, and DNA-based probes with fluorescent reporter and quencher, as well as the need for skilled workers, is one of the main problems. These instruments and dyes are not appropriate for use in low-resource lab settings due to a lack of funding [1–6].

With the use of a spectrophotometer, it is simple to obtain the spectra of white light shining on a certain material and measure the light that is reflected back from it. As a tool for measuring color, it ensure that the color is uniform. To separate the incident beam into its various wavelengths, a spectrophotometer is utilized. To modify the waves of a certain wavelength to fall on the test solution. It is a procedure through which the scientist or researcher determines the accuracy of the light source using a calibration standard. Make sure the tool work properly and right measurement is taken. Depending on the brand and make, several calibration techniques are used but on the other side spectrophotometers are highly expensive and bulky to make them unfeasible for remote and low resource lab settings [7–11].

Finally, Recombinase polymerase amplification (RPA) was used for colorimetric detection on Ercose device and for spectrophotometer that is easy to use, quick and sensitive was devised. The Ercose colorimetric data was compared with commercial spectrophotometer. It can serve as an alternate method for the detection of inorganic phosphate of amplified product because it is straightforward, quick, and easily adaptable to mass screening. The hue changed immediately as a result of the inorganic phosphate (Pi) ions’ reaction with the acidic molybdate to form the phosphomolybdate complex. Although the color can be qualitatively detected by the human eye, but a low-cost color sensor device Ercose has been developed for precise sample readings. The United States poses an important economic burden due to ovarian cancer screening, highlight the required inexpensive and reachable diagnostic approaches. Ercose is a cost effective as compared to spectrophotometer. The OVCAR3 cell line associate gene of TP53 sample was arranged for amplification. The RPA is less expensive, simple, time saving and not a laborious method as compared to other amplification techniques like PCR, on the other side spectrophotometer is an expensive device and not portable while Ercose is a simple, fast, portable and inexpensive device for low resources lab settings for colorimetric detection [12–14].

## RESULTS

### Inorganic Phosphate (Pi) based DNA analysis

The potential application of Pi achieved from amplified DNA. An inorganic pyrophosphate (PPi) is released by DNA polymerase during a dNTP addition process.

After an incubation time of 10 min., there was no Pi observed in the –ve control (Experiment A) and Pi detected in the R.mix (Experiment B) as was expected. However, in the absence of PPase, a slight greenish appearance in the yellow color was observed (Experiment C) which could possibly due to Pi contamination in some reagents possibly in dNTPs or primers. The false positive result problem was solved to use less amount of the magnesium ions related reagent and pyrophosphatase.

In the experiments (A to C) in Table 1, the incubation time was 10 min. So, in order to rule out the longer time related activity of PPase, further experiments were performed with virtually no incubation (0 Sec) or immediate addition of phosphate dye solution (PDS). A bright yellow color was observed in the –ve control (Experiment A) and green color was detected in the R.mix (Experiment B) as was expected. In the absence of PPase, again a light greenish appearance in the yellow color was observed (Experiment C) which could possibly due to Pi contamination. In experiment B, all required reagents were treated with amplified sample and achieved expected result in green color form. The only experiment B sample was tested on Ercose and spectrophotometer and compared for its validation. Experiments D and E in Table 2 also had an expected outcome.

**Table 1:** Detection of Pi in different reaction conditions after 10 min incubation

Color experiment	A	B	C
**Sample Type**	−VE Control	R.Mix	R.Mix – PPase
**Reaction conditions**	**10X Buffer** **dNTPs** **MgCl_2_** **PPase** **Klenow** **spDNA** Water 25°C (10 min) + PDS	10X Buffer dNTPs MgCl_2_ PPase Klenow spDNA Water 25°C (10 min) + PDS	10X Buffer dNTPs MgCl_2_ **PPase** Klenow spDNA Water 25°C (10 min) + PDS
**Color production**	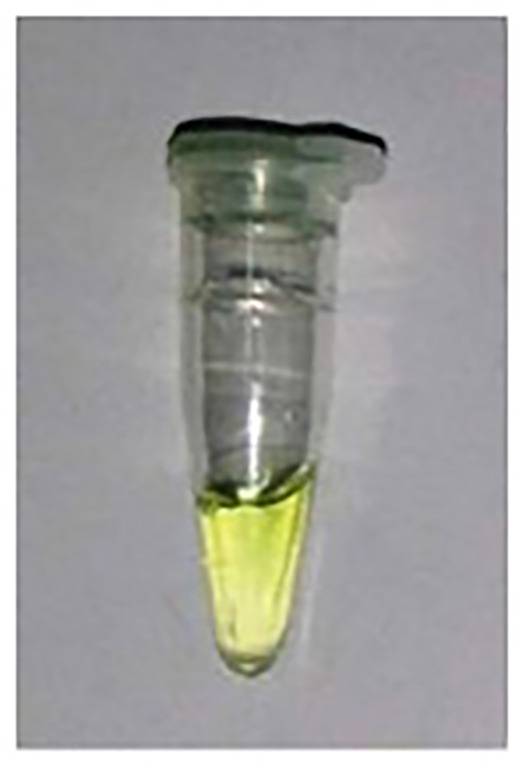	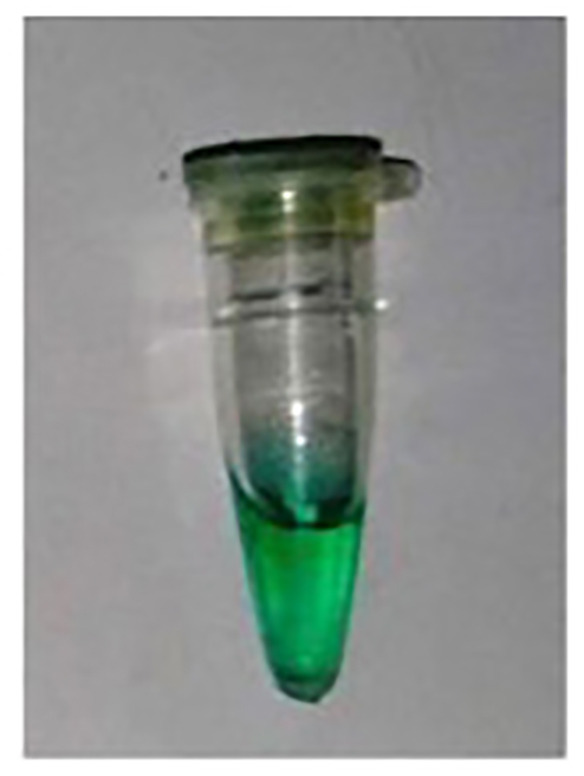	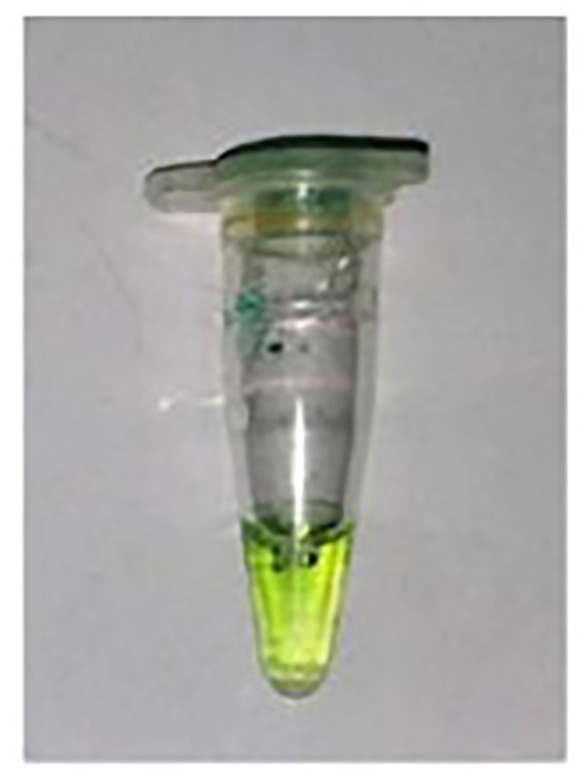
**Pi production**	No Pi	Pi ↑↑↑↑↑↑	Pi ↑
**Expected**	—	DNA Polymerase	DNA Polymerase
**Enzyme activities**	—	Pyrophosphatase (PPase)	—
**Observations**	As expected	As expected	Some reagent has Pi contamination (possibly dNTPs or primers)

**Table 2:** Detection of Pi in different reaction conditions after 0 Sec incubation

Color experiment	D	E	G
Sample Type	-VE Control	R.Mix	R.Mix – PPase
**Reaction conditions**	**Buffer** **dNTPs** **MgCl_2_** **PPase** **Klenow** **spDNA** **Water** 25°C (0 Sec) + PDS	Buffer dNTPs MgCl_2_ PPase Klenow spDNA Water 25°C (0 Sec) + PDS	Buffer dNTPs MgCl_2_ **PPase** Klenow spDNA Water 25°C (0 Sec) + PDS
**Color production**	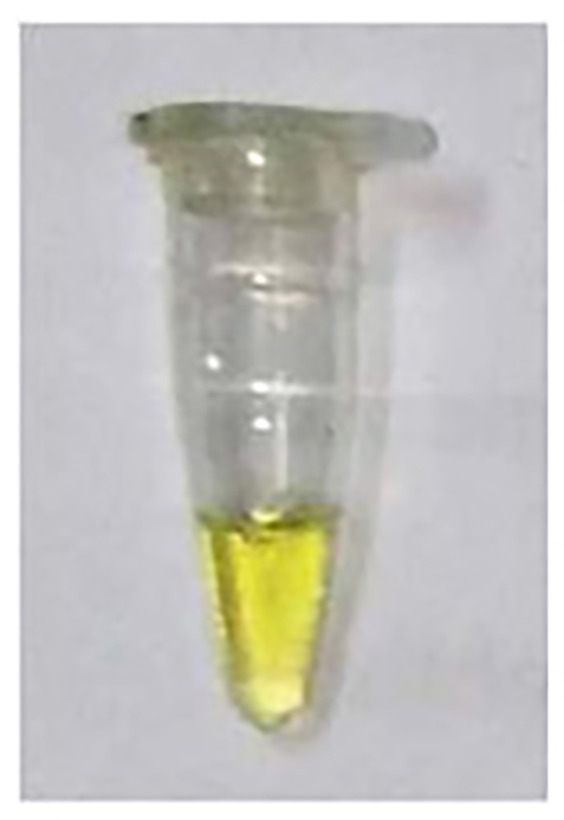	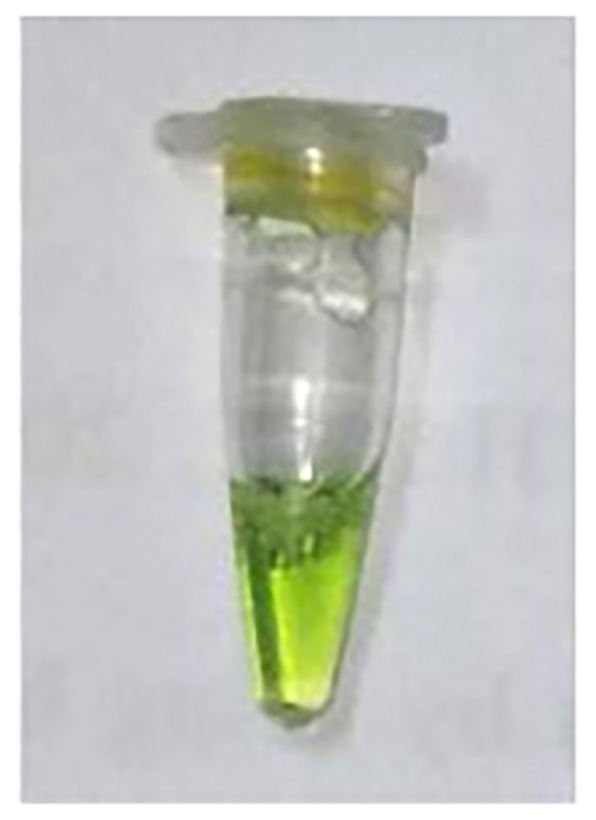	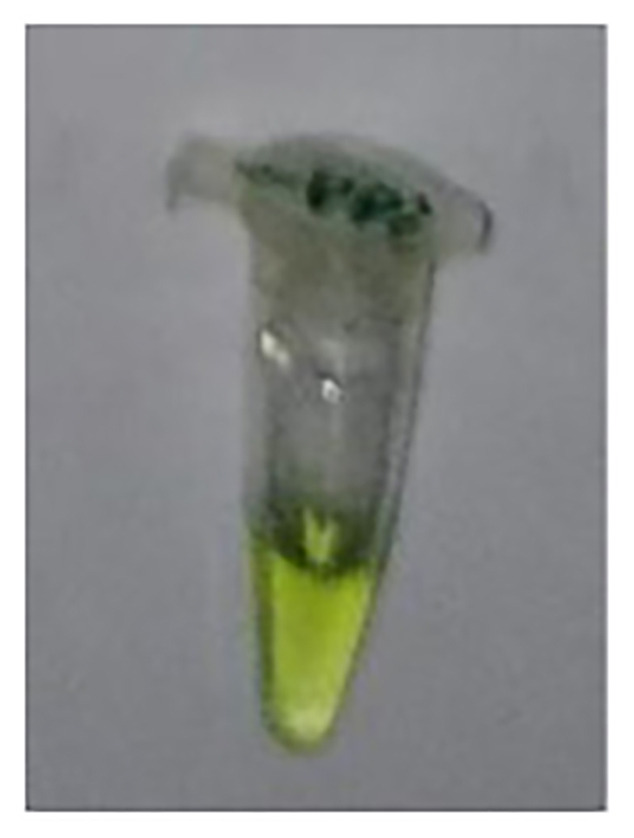
**Pi production**	No Pi	Pi ↑↑↑↑↑	Pi ↑↑
**Expected enzyme activities**	—	DNA Pol. Pyrophosphatase	DNA Pol.
**Observations**	As expected	As expected	No PPase so No dNTPase → possibly some reagent has Pi contamination

### Designing of colorimeter device Ercose using color sensor

**Figure 1:**
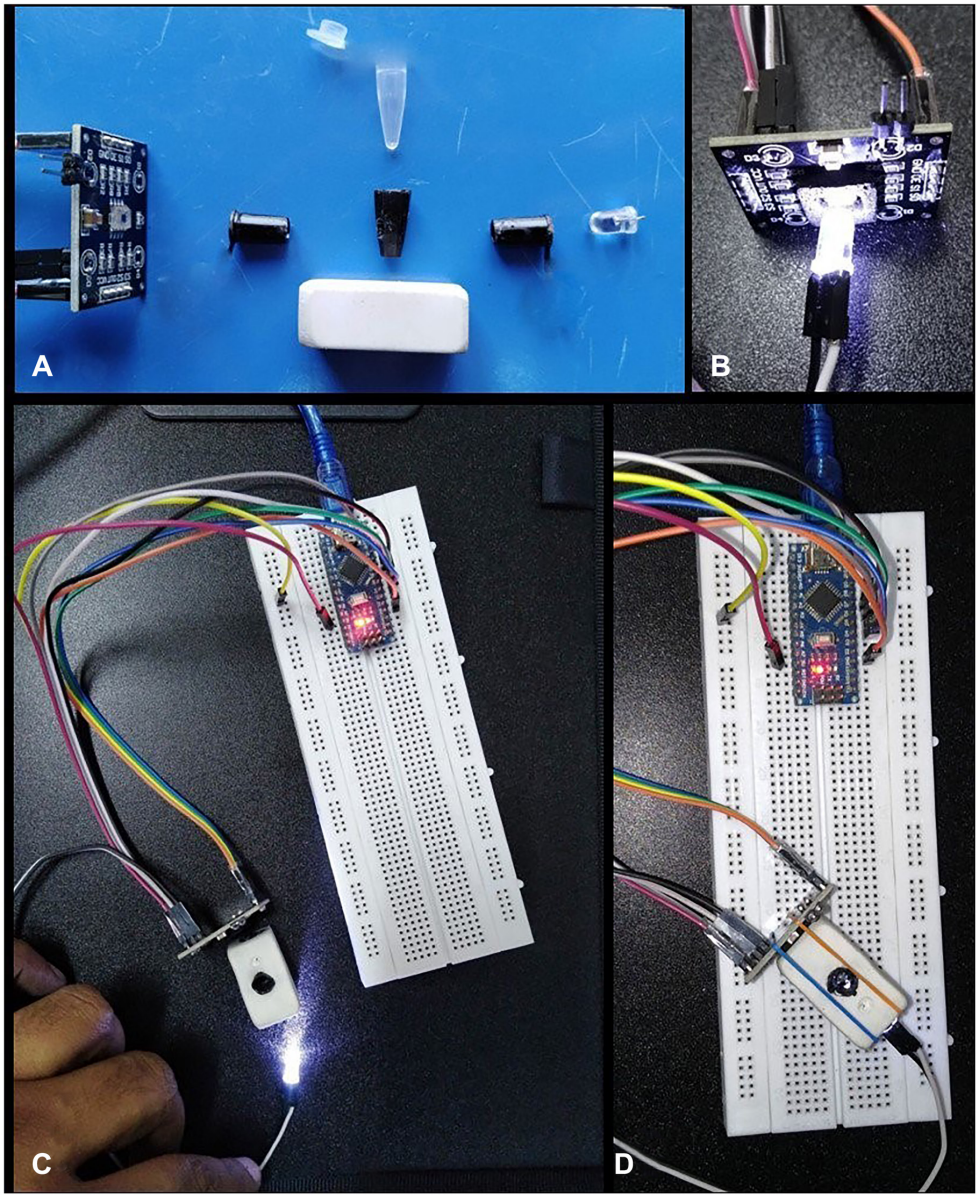
Color sensor based ercose colorimeter. (**A**) Different parts of Ercose colorimeter including color sensor TCS3200, LED, PDMS based sample holding tube (SHT) with covers, and last one is eraser. To made the holes on both side of the covers of color sensor TCS3200, LED and SHT the cross the LED light from SHT to color sensor and achieved the exact result, (**B**) Color sensor exposed with white light through white LED, (**C**) Ercose during assembly, (**D**) Ercose colorimeter in assembled form for testing the amplified HGSOC based Pi for green color readings. The two elastic rubber bands were used to fix the eraser with color sensor TCS3200, LED and SHT at one point.

The development of a colorimeter utilizing a color sensor allowed for the detection of minute differences between green colors that appear identical to the naked eye. White light from a white light emitting diode (LED) shines on the test solution in the tube, causing color to be formed. This color is then exposed to the color sensor (Figure 1), which produces readings of the colors as red: green: blue (RGB ratios). The arduino nano microcontroller was attached to the color sensor. RGB data was transmitted to a laptop through a USB link and entered into Microsoft Excel using the PLX-DAQ plug-in.

The developed colorimeter was optimized for detection of green color produced for pi-colorlock gold kit. For this purpose phosphate solutions of different concentrations were prepared starting from 100 with 10 parts reduction of Pi till 10. Data from Ercose color sensor device converted into normalized green and commercial spectrophotometer were obtained (Table 3) and compared to use it for calibration curve.

**Table 3:** Ercose and spectrophotometer Pi colorimetric detection data comparison

Pi concentration (μM)	Red	Green	Blue	Ercose data (Normalized green)	Spectrophotometer absorbance readings
100	25	94	29	0.368627	0.370
90	25	92	29	0.360784	0.362
80	23	92	29	0.360784	0.361
70	23	90	28	0.352941	0.353
60	23	88	25	0.345098	0.346
50	23	87	25	0.341176	0.344
40	22	84	25	0.329412	0.332
30	22	82	25	0.321569	0.325
20	19	80	25	0.313725	0.317
10	19	79	23	0.309804	0.314

When Pi concentration increases, the green value also increases, this is expected because malachite green forms a green colored complex with Pi. The relationship between Pi and normalized green is nearly linear between 10–100 μM. The Ercose values were scaled 0 to 1 by dividing 225 (max value in 8 bit sensor):


$$\text{Normalized Green}=\frac{\text{Raw Green}}{225}.$$

Beside the Ercose data is also compared with spectrophotometer for validation.

**Figure 2:**
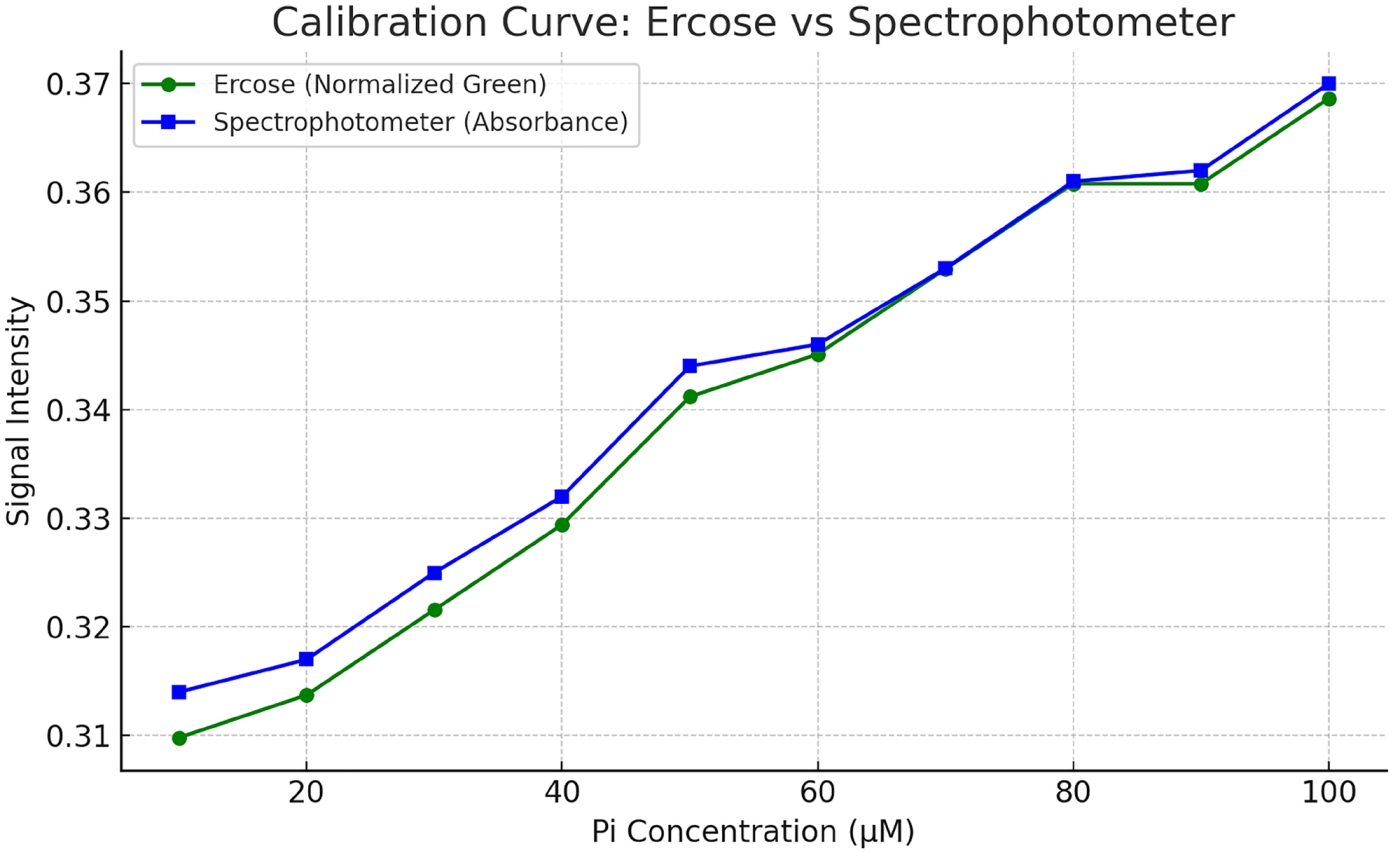
Calibration curve of Ercose vs. spectrophotometer. The Ercose sensor closely follows the spectrophotometer trend, indicating its reliability for low-cost detection.

**Figure 3:**
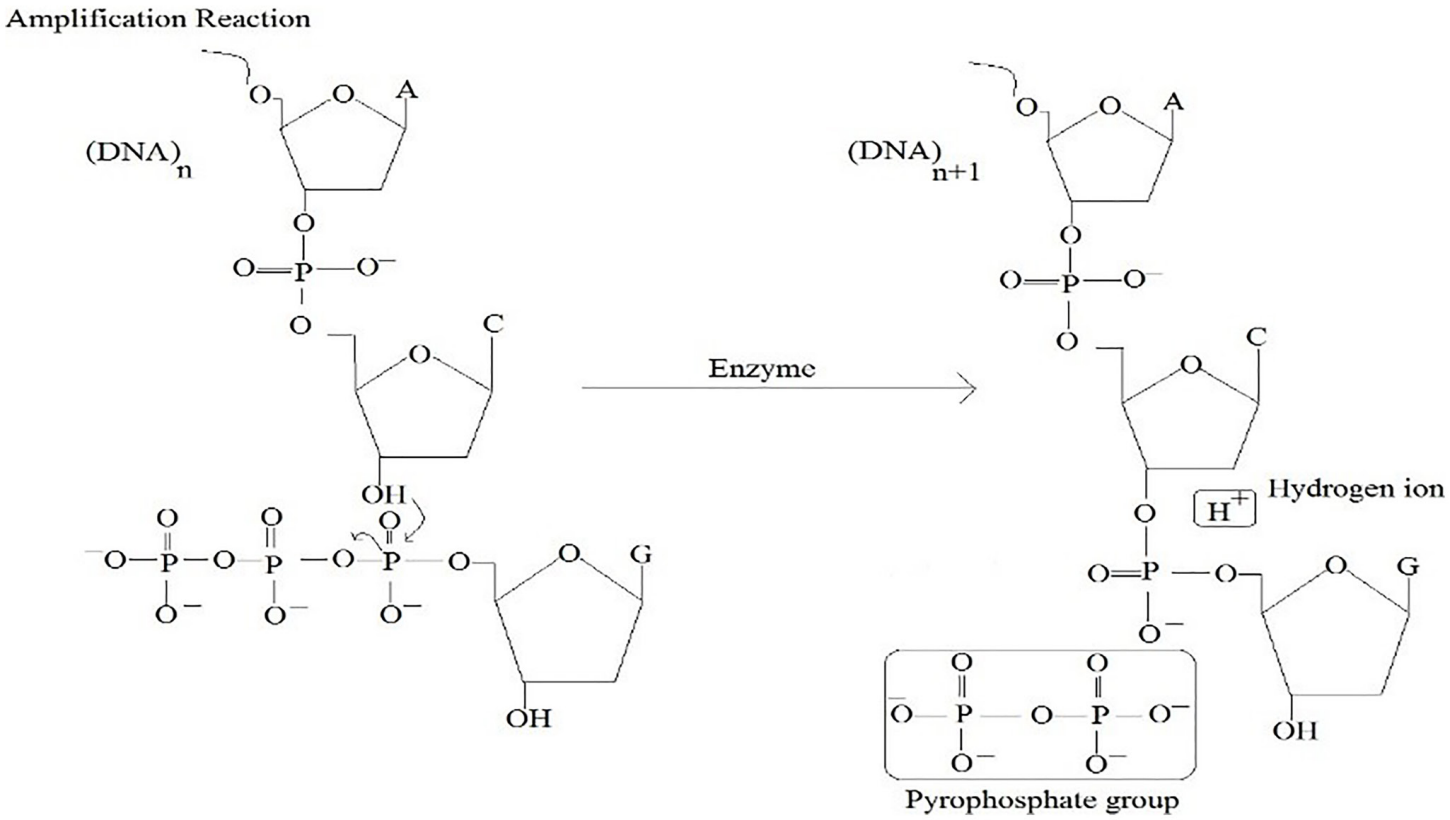
Amplification reaction in chemical structural form.

**Figure 4:**
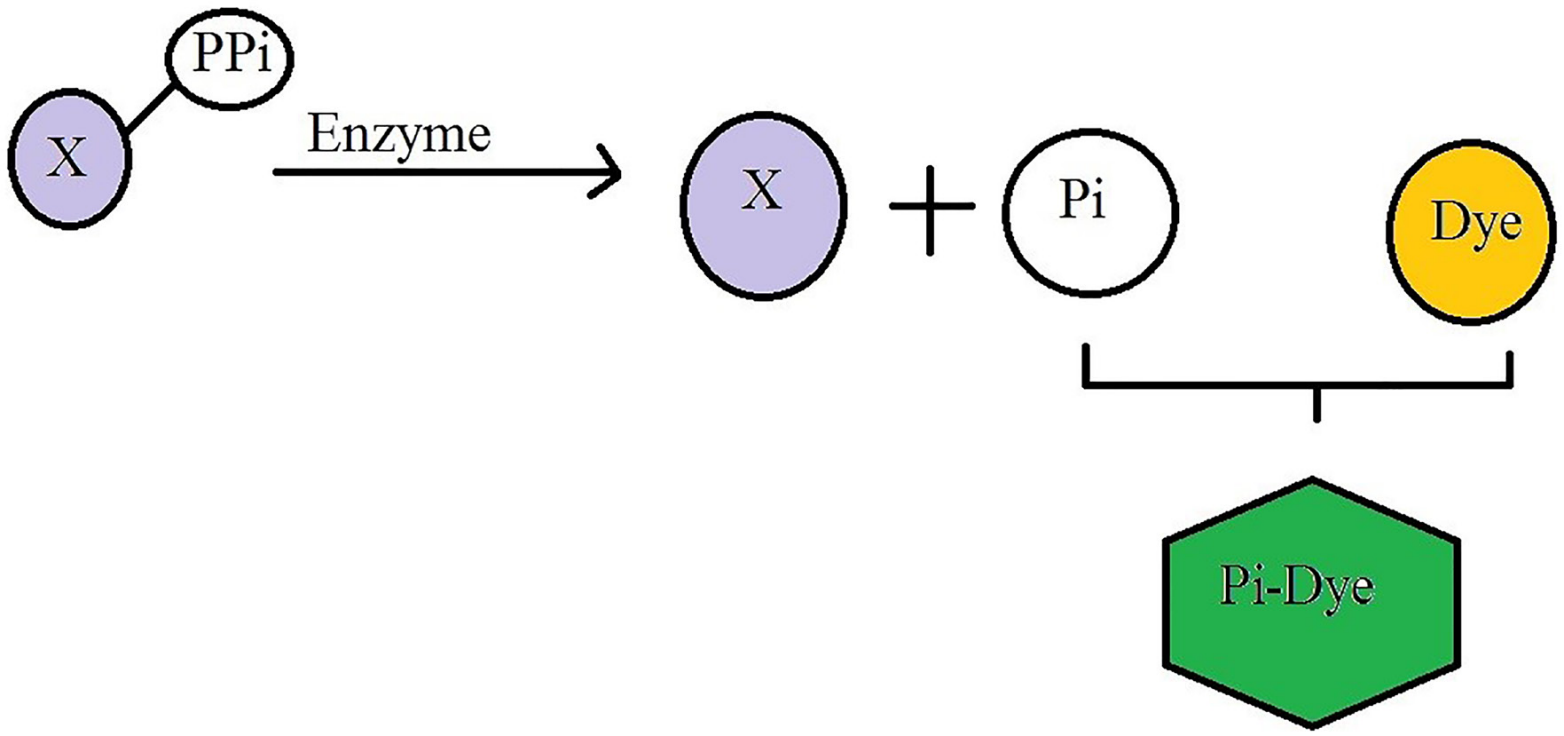
Principle of inorganic phosphate (Pi) dye-based detection: Pi is released from the enzyme substrate as a result of enzyme activity. The dye reagents (Orange), which turn green in the presence of Pi was added. The X was an amplified DNA sample that contained pyrophosphate (PPi), which was released after nucleotide bases were incorporated into the DNA template. It was subsequently reacted with the enzyme inorganic pyrophosphatase (PPase) to transform it into inorganic phosphate (Pi). PiColorLock^™^ dye reagents was react with inorganic phosphate (Pi) to turn the color in green.

The calibration curve shown in Figure 2 compares the signal responses of two detection methods. Ercose (Normalized Green) and the spectrophotometer (Absorbance), across a range of Pi concentrations from 10 to 100 μM. The green line with circular markers represents the Ercose sensor’s normalized green intensity, while the blue line with square markers indicates the spectrophotometric absorbance measurements. Both curves show a positive linear trend, indicating that as Pi concentration increases, the signal intensity for both methods also increases. This behavior confirms that both detection systems are sensitive to changes in Pi concentration. The spectrophotometer exhibits slightly higher readings across most concentrations, reflecting its greater sensitivity or possibly its ability to detect minor optical density differences. However, the enzymatic reaction played an important role in the conversion of PPi into Pi, as shown in Figures 3 and 4.

Notably, the Ercose system closely follows the spectrophotometer trend, suggesting its potential as a low-cost and reliable alternative for Pi quantification. The small differences in values between the two curves are consistent and fall within acceptable ranges, indicating that Ercose is a valid tool for phosphate measurement, especially in point-of-care settings where spectrophotometers are impractical.

## DISCUSSION

### Pi based colorimetric detection of HGSOC associated of OVCAR3 related TP53 gene

Creating a quick, easy-to-use detection system that is sensitive and reliable. For this reason, a quick, simple way to apply and measureable approach for visual detection of the amplified DNA is created. In order to extract the inorganic phosphate from the amplified genomic DNA, RPA was utilized as an amplification method. A yes or no answer can be distinguished with the naked eye thanks to a colorimetric change. It was considerably simpler for the observer to obtain a correct result using this method of Pi detection because the solution’s color changed from colorless to green in the presence of amplification. The picolorlock assay was based on the shift in malachite green dye in the presence of phosphomolybdate complexes, but unlike most malachite dye-based solutions, picolorlock gold delivers a stable end point signal and is not susceptible to precipitation. A unique stabiliser was included in Picolorlock to block nonenzymatic background noise. So, as the strand grew, nucleotides were added one by one during DNA amplification. As a result, pyrophosphate (PPi), which contains two phosphates, was released. The production of PPi would be hydrolyzed into Pi with the prior addition of inorganic pyrophosphatase (PPase). Visual detection was accomplished using only the human eye, and precise reading of samples containing Pi was accomplished using a color sensor device Ercose.

Therefore, there was a critical need to create a detection system that was quick, easy, useful and portable in addition to being sensitive and dependable. For this reason, visual detection may be used as an additional or alternative approach to detect the amplified DNA in Pi form. Visual detection has the advantages of being quick, direct and easily applicable to mass screening. The Pi was detected after amplification using the colorimetric technique and color sensor device Ercose. This approach is easy, straightforward and economical. There are no health-related issues with this procedure. The HGSOC amplified sample with picolorlock reagent is specifically designed to provide sensitive Pi detection.

### Ercose device with real time data acquisition and analysis

The Ercose was performed in the dark due to color sensor (TCS3200) sensitivity from light. The eraser was used to give the control light environment for the sample and color sensor device to achieve the required result. Breadboard and arduino pin connections are made using jumper wires. Arduino nano is a flexible, compatible and breadboard friendly microcontroller board that was designed by Arduino.cc in Italy based on ATmega328p (Version 3.0). It has precisely the same capability as arduino UNO but is much smaller in size. The arduino nano can be programmed with the use of arduino software, and it can be controlled via programming. The PLX-DAQ plugin (Parallax Inc., Rocklin, CA, USA) for Microsoft Excel (Microsoft Corporation, Redmond, WA, USA) was used to show real-time data that was transmitted to a computer over a universal serial bus (USB). Alternately, the data can be shared on a smart phone using any data plot application built on an arduino nano. The information collected, stored and distributed by the data collecting system regarding each nucleotide base integrated during DNA synthesis proved very useful. The system offers a full environment for technological interaction and information production, allowing users to see results in real time.

### Comparison color sensor with photomultiplier tube (PMT)

A photomultiplier tube (PMT) is an electrical apparatus that amplifies the energy of the photons that strike it into stronger electrical impulses in order to detect weak electromagnetic radiation, often light. The PMT is widely recognized as the industry standard for spectrophotometry in research, including when employing open-surface or digital micro-fluidics, as well as in commercial equipment. A few of the tradeoffs include the mechanical brittleness of PMTs, the requirement for an extraordinarily stable high voltage power supply, the need for cooling to reduce signal-to-noise ratio, the larger size with fewer shape and size options, the susceptibility to external magnetic fields, and the high cost. Although it has been used as an alternative to the PMT, the color sensor has not been standardized for spectrophotometers. The color sensor is far more user-friendly and inexpensive than a PMT.

### Detection capability of color sensor

The picolorlock gold reagent with Pi produces the strongest signal at 635 nm, however it is feasible to attain great sensitivity throughout a wide spectrum of wavelengths (590–660 nm). The visual detection method based on the Pi is more stable and doesn’t require any custom labels. The wavelengths are 700 nm. When a color filter is chosen for the TCS3200, it only permits one primary color to pass through while blocking the passage of other primary colors. For instance, when the green filter is chosen, only the green portion of the incident light can through; the blue and red portions are blocked, so that the light intensity of the green light can be obtained. Likewise, by using different filters, one may determine the light intensity of red and blue light. Examine the hue of the light reflected on the TCS3200 sensor using these three values. For colorimeter measurement uses like medical diagnostics, the programmable color light-tofrequency converter TCS3200 is appropriate. White balance is used to define white to the system in terms of color recognition. Although the proportions of the three fundamental hues in white are not quite equal, in theory, white is composed of the same amount of red, green and blue. The RGB output of the TCS3200 light sensor is not equal because of the light sensor’s varying sensitivity to these three primary hues. Therefore, it is required to correct the white balance before to testing in order for the TCS3200 to be equally sensitive to the three primary colors in the detected “white.” In order to recognize colors afterwards, the white balance is adjusted. The device’s specific procedures and techniques for adjusting white balance are as follows. In order for the incident light to reach the TCS3200, place the empty test tube above the sensor and a white light source above the test tube. The red, green, and blue filters are chosen one at a time, and the red, green, and blue values were measured separately. The three adjustment parameters can be determined using the above-described procedure.

### Circuit diagram of TCS3200 color sensor module

The TCS3200 color sensor’s circuit schematic is displayed below and is very straightforward and simple to comprehend. A 1K resistor is connected in series with the TCS3200 in the schematic diagram, which also shows 4 LEDs and a 330R current-limiting resistor. This is because the out pin of the integrated circuit has a low impedance. S0 and S1 are being pulled up by two 10K pull-up resistors. A 10uF filter capacitor and a 100nF decoupling capacitor are also present on the board, along with two 0 ohm jumper links.

### Drawbacks of Ercose device and improvement strategies

The biggest drawback of the Ercose device is open source, the present form of device was fabricated with different electronics and 3D printed parts. The goal in developing the current design was to demonstrate a color sensor device as proof of concept. To enhance the device future design and functionality, certain tactics can be recommended. The future simple strategy for GUI will be without universal serial bus (USB) to directly connect with cell phone via bluetooth for real time analysis and graphical plotting.

### Sensitivity

Color sensors and samples must be read at a constant temperature because they are sensitive to light and temperature. The experiments were carried out at 21–24°C in a control lab environment with regulated lighting and temperature to get around the color sensor issue. The operating range of the TCS3200 color sensor is −40 to 70°C. Most inline color sensors currently in the market are classified as having an accuracy of ΔE of 1–1.5, which is comparable to performance of the human eye. Modern color sensors, however, provide a ΔE of 0.8 at reasonably low-cost levels. People will describe the same hue differently, despite the fact that the human eye is highly good at discriminating colors. For this purpose, used a properly calibrated color-sensing device Ercose to quantify color. The TCS3200 color sensor sensitivity is 0.1 lux to 10,000 lux for light range detection, minimum color difference detection is 5–10% and color resolution is 8 bit per channel.

### Cost distribution

The color sensor device used straightforward, widely accessible and extremely affordable components to deliver results that were quite reliable. With a cost comparison of roughly $100200 for PMT while complete spectrophotometer is very expensive, the excellent performanceto-cost ratio of color sensor under $6 may be appreciated as being the cheapest color sensors in the market worldwide. The other parts are a microcontroller ($7.5), a 9 V battery ($1.3), an eraser, jumping wires, and other electronic parts ($4.5). A 3D printed components was estimated to cost $2 and a laser-cut component to cost about $1.2. Other costs include nucleic acid sample and primers ($5.5), RPA (including, DNA polymerase, dNTPs other reagents, etc.) considering a single reaction ($4.5) and pyrophosphatase ($1). For color reagents, the reagent cost per experiment was $2. The proof-of-concept color sensor device and reagents cost about $35 to $40 in total, which is substantially less expensive for a device like a spectrophotometer. This makes the color sensor device Ercose practical in environments with limited resources.

## MATERIALS AND METHODS

### Development of color sensor device

A color sensor device was created using sensor and control electronic, jumper wire, a breadboard, sample holding tube (SHT) and its cover, color sensor and white LED covering tubes and eraser.

#### 3D printing

Solidworks (Dassault, France) is a computer aided design (CAD) and computeraided engineering (CAE) program for solid modelling that was used to create the covers of SHT, color sensor and LED with resin. PreForm software (Formlabs, USA) was used to prepare design files for printing after importing them in the STL format. The designs were then printed using a Form 2 printer (FormLabs, USA), which utilizes stereolithography (SLA)-based 3D printing to create solid, isotropic parts from a liquid photopolymer resin using a 405 nm laser. While PDMS based SHT was arranged with neighboring lab. PDMS is an optically transparent substance that is typically inert in aqueous settings.

#### Finishing

The printed part underwent the following processes to succeed the essential mechanical properties: (i) support structure removal with a cutter plier; (ii) IPA (Iso-propyl alcohol) rinsing for 8–10 minutes on an instrument like Form Wash (Formlabs, USA); (iii) postcuring with UV light for 12–15 minutes at 60°C on a Form Cure instrument (Formlabs, USA); and (iv) sanding to smoothen the rough.

#### Laser cutting

The LN-1390 CO_2_ laser engraver, China, was used to cut clear edges of the covers of SHT, color sensor and LED. All the covers were cut with laser engraver according to the requirement.

#### Eraser and jumper wires

The eraser is an easily available thing that was used to give the control light environment for the sample and color sensor device to achieve the required result. Connections between breadboard and Arduino pins are made using jumper wires. Wire up all the circuits with these.

### Sensing/control electronics

#### Color sensing

The color sensor (TCS3200) was utilized to sense the color in order to identify the DNA amplified based Pi green color. After development, all covers of SHT, color sensor and LED were pasted with black tape to produce the control environment for experiment.

#### Control electronics

All of the inputs and outputs needed for the color sensor device Ercose were managed by an arduino nano (Arduino, Italy) microcontroller. The analogue and digital pins of a microcontroller were used to connect the color sensor. With the help of the arduino IDE (Integrated Development Environment), arduino nano was programmed.

#### Data gaining and study

The data from the Pi color sensor was transferred through a USB connection to a computer and shown using the PLX-DAQ plugin (Parallax Inc., Rocklin, CA, USA) in Microsoft Excel (Microsoft Corporation, Redmond, WA, USA). Data was transformed into a graph for data analysis.

### Samples and primer design for RPA amplification

The high grade serous ovarian carcinoma (HGSOC) for TP53 gene sample, and primers were arranged from (IDT, USA). Using the NCBI program, primers were created, with product length 123 bp. The primer was 20 bp in length. The position of the primers was 5–3, with the forward primer (IDT, USA), CCTGGGTGTAGATGATGGGG, and the reverse primer (IDT, USA), CGGGTGGATGTGCAAAGAAG.

### RT (Reverse transcription) reaction

Before starting the RT process, 5 μg of total RNA in a 10 μl volume was heated for 5 to 10 minutes at 65°C before being cooled on ice. The following component was set up in a 1.5 ml eppendorf tube then 10.0 μl denatured RNA was warmth, 3.0 μl (10x) PCR buffer (Invitrogen), 2.5 μl (10 mM) dNTPs (Biocompare), 6.0 μl (25 mM) MgCl_2_ (Sigma-Aldrich), 1.0 μl (1.5 μg) random primers (Invitrogen), 0.5 μl (200 U/μl) SuperScript II reverse transcriptase (Invitrogen) and 17.0 μl water (Sigma-Aldrich) was used for reverse transcription reaction. The sample was incubated at 42°C for an hour after being left at 25°C for 10 minutes. The cDNA was chilled after being denatured at 95°C.

### RPA assay

Recombinase polymerase amplification (RPA), an isothermal amplification method that operates at 37°C to 42°C, is an alternative to the PCR method. RPA just required a very small number of chemicals and equipment. A 5 μl cDNA product was used for RPA reaction by using the TwistAmp^®^Basic Kit (TwistDx^™^). The concentration of reverse primer was 2.4 μl (10 μM, IDT, USA) and forward primer was 2.4 μl (10 μM, IDT, USA), but the rehydration buffer was 29.5 μl, nuclease free water 12.2 μl (Sigma-Aldrich) and magnesium acetate was 2.5 μl (280 mM) total volume was 54 μl. Positive control was executed to test the activity of kit components. The positive control primer mix was 6 μl, rehydration buffer 29.5 μl, positive control template 10 μl and magnesium acetate was 2.5 μl (280 mM). The template strand for the positive control was 143 bp. The replacement of cDNA and all other reagents with nuclease-free water was used as a negative control. At 42°C, the RPA experimental reaction was carried out in a water bath at JOANLAB in China. RPA’s ability to do tasks without the need for expensive equipment is one of its primary advantages.

### Detection of inorganic phosphate (Pi)

The Picolorlock kit (Innova Biosciences) was used for a colorimetric approach. The HGSOC amplified sample 9 μl was utilized to detect inorganic phosphate (Pi), the test was quick and simple to carry out, and it also required minimal chemicals and equipment. After amplification reaction, the nuclease free water was added 2.8 μl with inorganic pyrophosphatase (NEB) 2 units of 0.2 μl was used to hydrolyze the pyrophosphate (PPi) into inorganic phosphate (Pi). Follow the sample to prepare the reagents as a picolorlock gold was 15 μl, stabilizer was 6 μl and accelerator was0.6 μl. Total volume was 33.6 μl in each PCR tube to add 20.4 μl distilled water to made it 54 μl for colorimetric detection, in negative control and PPase skipped reagent described in Tables 1 and 2 replace with distilled water to make total volume 54 μl.

### Procedure

Accelerator and Picolorlock Gold were combined in this experiment. Accelerator aids in accelerating the reaction while the picolorlock gold aids in changing the reaction’s color. The HGSOC amplified sample was combined with the picolorlock gold and accelerator after amplification, which caused the solution’s color change to green. These reagents changed color when Pi was present, and the amount of Pi released from a substrate may be determined using a straightforward absorbance reading. However, remember to add the stabiliser last. Avoid mixing the accelerator and Picolorlock Gold immediately with the stabiliser. The picolorlock assay is based on the change in malachite green dye absorbance in the presence of phosphomolybdate complexes, however in contrast to most solutions based on malachite dye, picolorlock gold provided a stable end point signal and it was not easily prone to precipitate.

### Negative control

A negative control was essentially an experiment carried out concurrently with a major experiment using identical methods, with the exception that the treatment was altered to something that was expected to have no result. Negative control solution was 54 μl with nuclease free water 2.8 μl and inorganic pyrophosphatase (NEB) was 2 units of 0.2 μl that was used to check the inorganic phosphate with the help of picolorlock reagents, picolorlock reagents was used 15 μl picolorlock gold, 6 μl stabilizer but the accelerator was 0.6 μl, used the distilled water to complete the total volume 54 μl.

## CONCLUSIONS

To demonstrated a very simple, cost effective, portable and easy to operate color sensor device Ercose for the analysis of HGSOC based Pi using TCS3200 color sensor, eraser and PDMS based SHT and also 3D printed covers pasted black tape for color sensor, SHT and for white LED, to protect the sample from the open environment and achieved the required result. The most durable detectors on the market that are operate with very basic circuits are TCS3200 color sensor. In comparison to PMT, color sensor function are far better when dealing with background noise. In circumstances with little resources and little training, such a device Ercose is very helpful for rapid detection and accurate analysis by using a small stretch of amplified nucleic acid of Pi for colorimetric detection, especially for resource poor settings.
